# Supplementary material for: Historical and projected future range sizes of the world’s mammals, birds, and amphibians
Source: Nat Commun. 2020 Nov 6;11:5633. doi: 10.1038/s41467-020-19455-9 (PMC7648644; doi:10.1038/s41467-020-19455-9)
Supplement: Supplementary file 1 — Supplementary Information [file 41467_2020_19455_MOESM1_ESM.pdf]

Supplementary Information

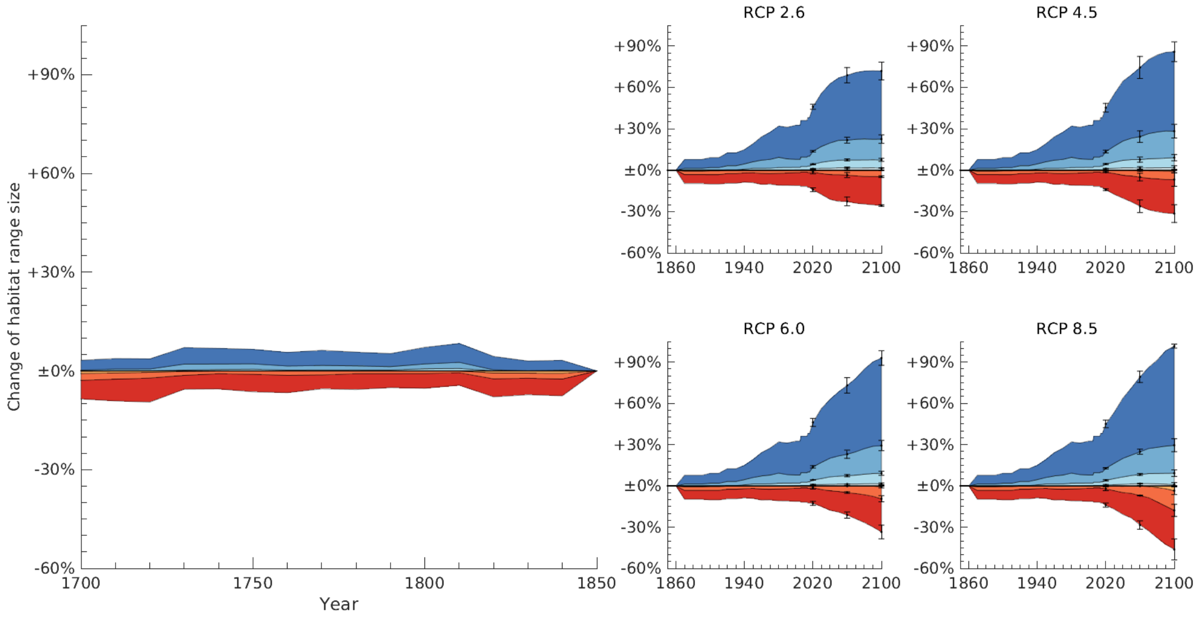

Supplementary Figure 1. Past and RCP-specific future changes in range sizes through time based only on climate-driven biome distributions (i.e. in the hypothetical absence of anthropogenic land use), relative to potential natural range sizes in 1850. Colour shades and the black line represent 10–90% percentiles and the median of range changes across all species, respectively (analogous to Fig. 1a). Results for the future represent the means of projections derived based on n=3 different climate models; uncertainty bars represent standard deviations, and indicate the uncertainty of the projections with respect to the climate data (Methods).

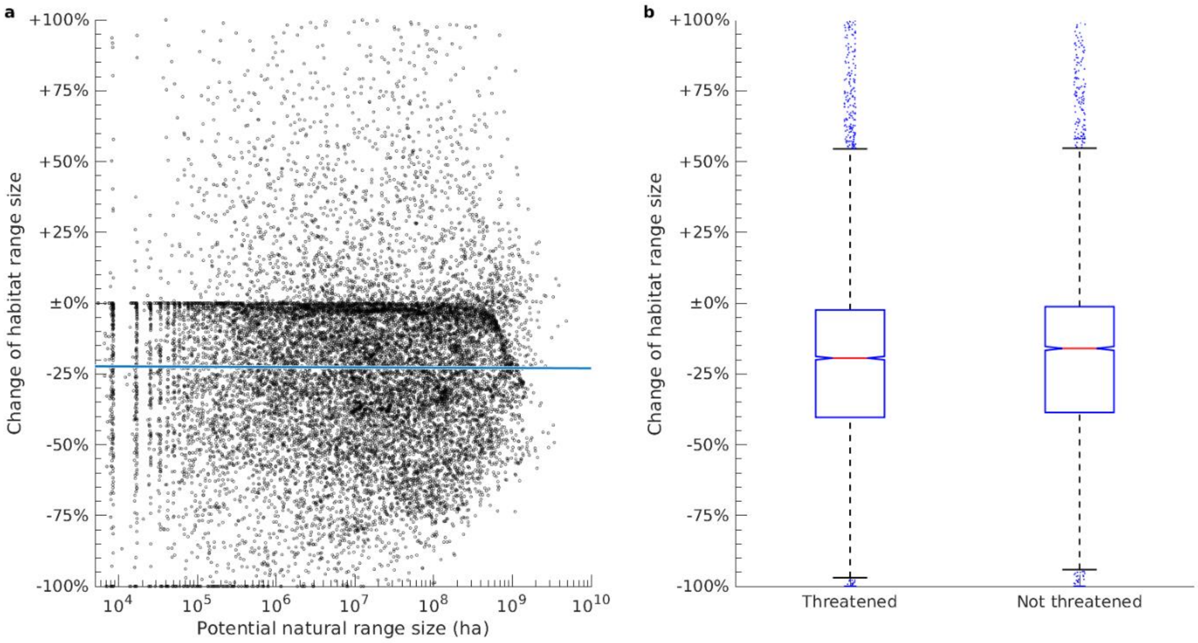

Supplementary Figure 2. Range changes in small-ranged and threatened species. **(a)** Changes of range sizes in 2016 (based on the HYDE 3.2 baseline land use), relative to potential natural range sizes in 1850, against potential natural range sizes in 1850. The linear coefficient of the fitted robust (using a bisquare weight function) log-linear relationship (blue line) is not significantly different from 0 based on a two-sided t-test (p=0.95). **(b)** Change of range sizes in 2016 of threatened and non-threatened species. Threatened species include those classified as vulnerable, endangered, and critically endangered in 2016. Red lines represent medians, blue box limits represent upper and lower quartiles, black whiskers represent 1.5×interquartile range, blue dots represent outliers (n=16,919 species). Median range loss for threatened species is not significantly different from zero (two-sided t-test, p=0.9). For visualisation purposes, y-axes in (A) and (B) were capped at +100% range size increases.

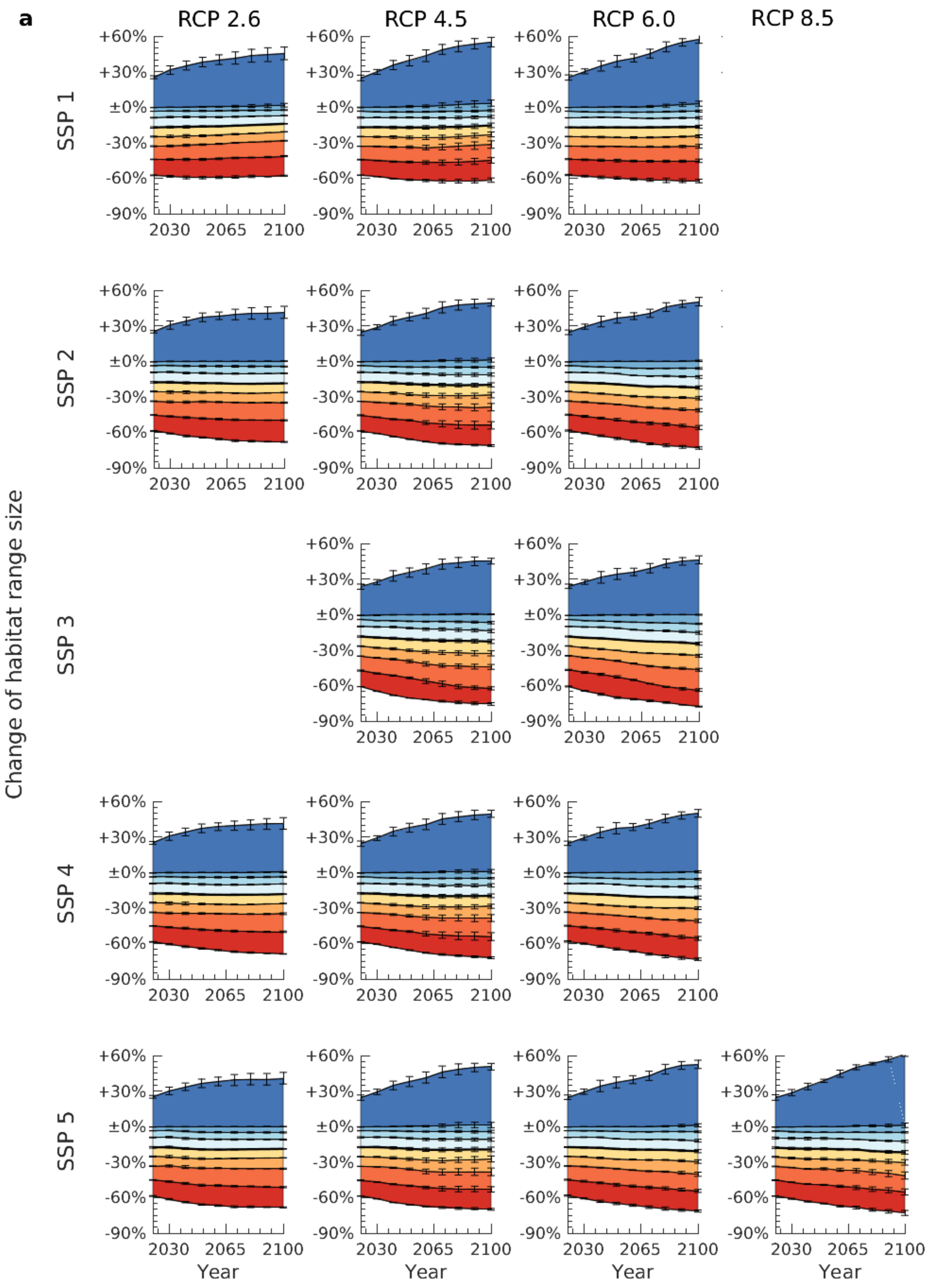
a
RCP 2.6
RCP 4.5
RCP 6.0
RCP 8.5
SSP 1
SSP 2
SSP 3
SSP 4
SSP 5
Change of habitat range size
+60%
+30%
±0%
-30%
-60%
-90%
2030
2065
2100
Year

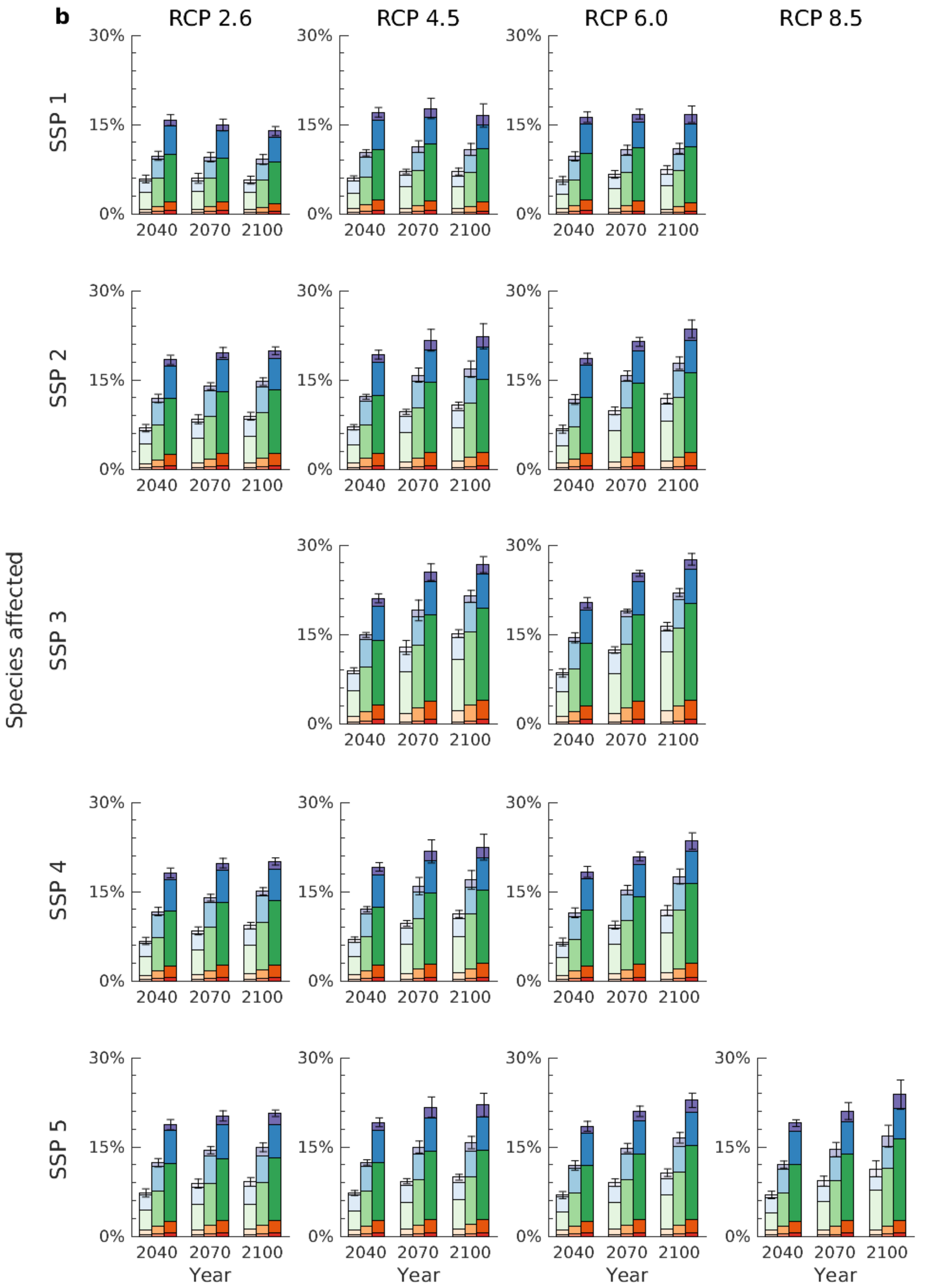

Supplementary Figure 3. Projected future changes in species' range sizes. **(a)** Future SSP- and RCP-specific changes in the habitat range sizes between 2025 and 2100 CE, relative to potential natural range sizes in 1850. Colour shades and the black line represent 10–90% percentiles and the median of range changes across all species, respectively. **(b)** Percentages

of species affected by critical range losses, broken down by species' primary mega-biome. Lines and coloured areas in (a) and bar charts in (b) represent the means of projections derived based on n=3 different climate models; uncertainty bars represent standard deviations, and indicate the uncertainty of the projections with respect to the climate data (Methods). Colour schemes in (a) and (b) are identical to those used in Figs. 1a and 1b, respectively.

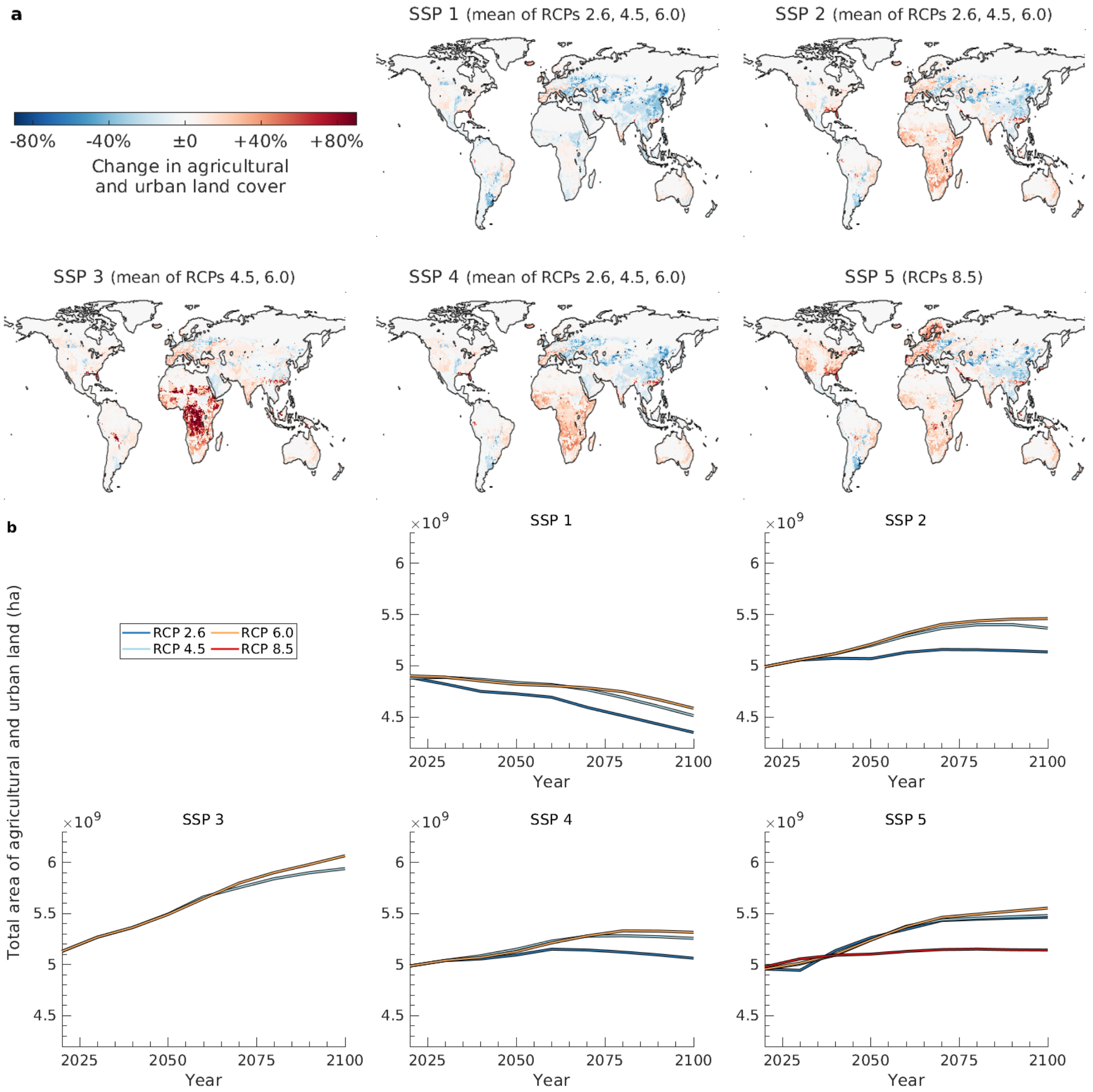

Supplementary Figure 4. Overview of future land use scenarios. **(a)** Difference between SSP-specific distributions of agricultural and urban land in 2100 (averaged across the RCPs available for the relevant SSP) and the current (year 2016) distribution. **(b)** SSP- and RCP-specific trajectories of the total area of converted land between 2020 and 2100.

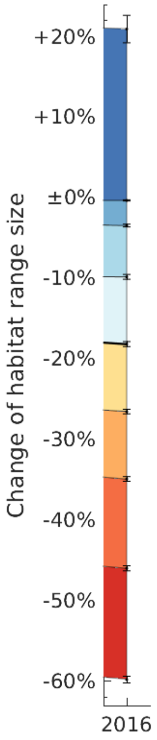

Supplementary Figure 5. Means and standard deviations of the 10–90% percentiles of species range changes in 2016 (cf. far right end of Fig. 1a) obtained from n=10^4 bootstrapping resamples of species-specific range sizes (Methods). The underlying land use scenario is the HYDE 3.2 baseline.

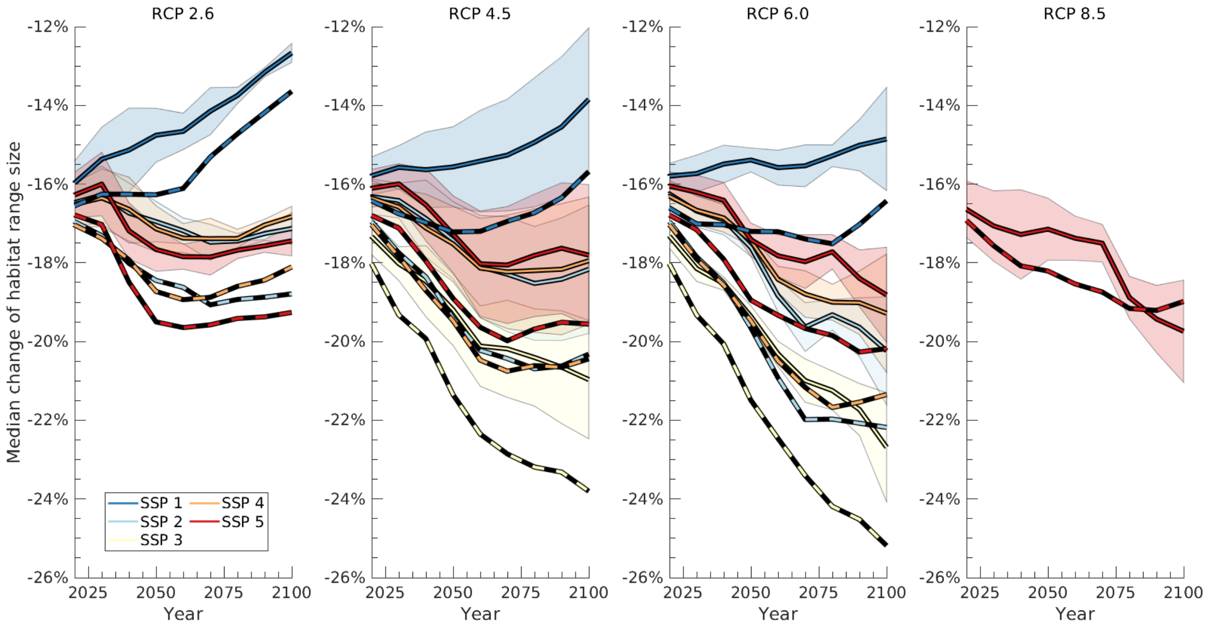

Supplementary Figure 6. Projected future changes in range sizes assuming no future biome changes. Solid lines and uncertainty bands are identical to those in Fig. 3, representing the default scenario of shifts in global biome distributions in response to climate change (Methods). Dashed lines represent the scenario in which the global biome distribution is set to be identical to the one estimated for the year 2016 at all future points in time, i.e. range sizes are driven only by land use. Land use projections are the same as those used in Fig. 3.

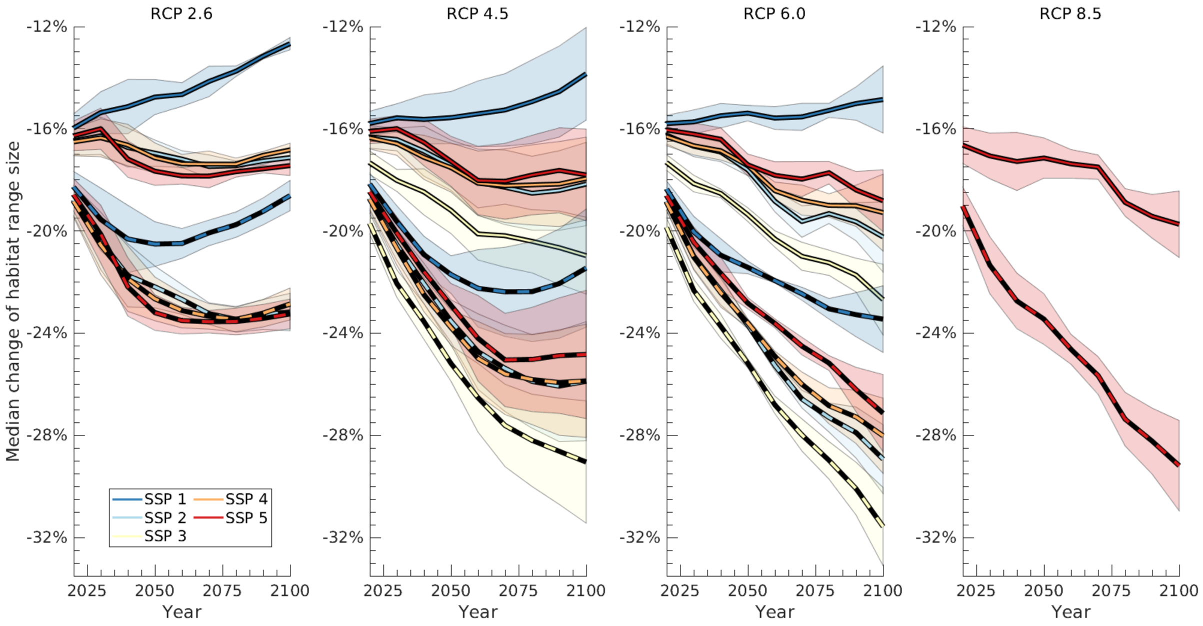

Supplementary Figure 7. Projected future changes in range sizes when assuming that species' future habitat ranges do not extend beyond current potential natural ranges. Solid lines and the corresponding uncertainty bands are identical to those in Fig. 3, representing the default scenario of areas being counted towards a species' habitat range if they lie within the species' extent of occurrence and represent suitable habitat under the appropriate climate and land use scenario. Dashed lines and the corresponding uncertainty bands represent means and standard deviations (across n=3 different climate models), respectively, based on the scenario in which areas are counted towards a species' habitat range only if they represent geographically and climatically suitable habitat both at the relevant point in the future and at present day (year 2016). Thus, suitable areas are excluded if the species is not already present in them, i.e. would have to migrate there. Land use projections are the same as those used in Fig. 3.

**BIOME4 habitat categories**	**Equivalent IUCN species habitat categories**	**Mega-biome**
Desert, Barren	Desert	Desert
Cold mixed forest<br>Evergreen taiga<br>Deciduous taiga	Boreal forest<br>Subarctic forest<br>Subantarctic forest	Boreal forest
Tropical evergreen forest<br>Tropical semi-deciduous forest<br>Tropical deciduous forest	Subtropical forest<br>Tropical forest	Tropical forest
Temperate deciduous forest<br>Temperate conifer forest<br>Warm mixed forest<br>Cool mixed forest<br>Cool conifer forest	Temperate forest	Temperate forest
Boreal parkland<br>Steppe tundra<br>Dwarf shrub tundra<br>Prostate shrub tundra<br>Cushion forb lichen moss tundra	Subarctic grassland<br>Subantarctic grassland<br>Tundra	Tundra
Tropical grassland	Subtropical grassland<br>Tropical grassland	Grassland and dry shrubland
Temperate grassland	Temperate grassland	Grassland and dry shrubland
Tropical savanna<br>Temperate broadleaved savanna	Savanna	Dry woodland and savanna
Shrub tundra<br>Drawf shrub tundra<br>Prostate shrub tundra<br>Cushion forb lichen moss tundra	Boreal shrubland<br>Subarctic shrubland<br>Subantarctic shrubland	Tundra
Temperate sclerophyll woodland<br>Open conifer woodland	Mediterranean-type shrubby vegetation	Dry woodland and savanna
Tropical shrubland	Tropical shrubland	Grassland and dry shrubland
Temperate sclerophyll shrubland	Temperate shrubland	Grassland and dry shrubland
**HYDE and AIM land use categories**		
Cropland	Arable Land	
Pasture	Pastureland	
Urban	Urban areas	

Supplementary Table 1. Matching between land cover types in the global land use and BIOME4 data, and IUCN categories of species habitats. Species-specific primary mega-biomes were assigned based on the mega-biome shown in the third column that accounts for the largest area of the species' potential natural range in 1850.
