# Supplementary material for: Historical and projected future range sizes of the world’s mammals, birds, and amphibians
Source: Nat Commun. 2020 Nov 6;11:5633. doi: 10.1038/s41467-020-19455-9 (PMC7648644; doi:10.1038/s41467-020-19455-9)
Supplement: Supplementary file 2 — Description of Additional Supplementary Files [file 41467_2020_19455_MOESM2_ESM.pdf]

Description of Additional Supplementary Files

Title: Supplementary Movie 1.

Description: Global biome distributions between 1700 and 2016.

Title: Supplementary Movie 2.

Description: Global biome distributions between 2020 and 2100 based on RCP 2.6 and the CSIRO-Mk3.6.0 climate model.

Title: Supplementary Movie 3.

Description: Global biome distributions between 2020 and 2100 based on RCP 2.6 and the HadGEM2-ES climate model.

Title: Supplementary Movie 4.

Description: Global biome distributions between 2020 and 2100 based on RCP 2.6 and the MIROC5 climate model.

Title: Supplementary Movie 5.

Description: Global biome distributions between 2020 and 2100 based on RCP 4.5 and the CSIRO-Mk3.6.0 climate model.

Title: Supplementary Movie 6.

Description: Global biome distributions between 2020 and 2100 based on RCP 4.5 and the HadGEM2-ES climate model.

Title: Supplementary Movie 7.

Description: Global biome distributions between 2020 and 2100 based on RCP 4.5 and the MIROC5 climate model.

Title: Supplementary Movie 8.

Description: Global biome distributions between 2020 and 2100 based on RCP 6.0 and the CSIRO-Mk3.6.0 climate model.

Title: Supplementary Movie 9.

Description: Global biome distributions between 2020 and 2100 based on RCP 6.0 and the HadGEM2-ES climate model.

Title: Supplementary Movie 10.

Description: Global biome distributions between 2020 and 2100 based on RCP 6.0 and the MIROC5 climate model.

Title: Supplementary Movie 11.

Description: Global biome distributions between 2020 and 2100 based on RCP 8.5 and the CSIRO-Mk3.6.0 climate model.

Title: Supplementary Movie 12.

Description: Global biome distributions between 2020 and 2100 based on RCP 8.5 and the HadGEM2-ES climate model.

Title: Supplementary Movie 13.

Description: Global biome distributions between 2020 and 2100 based on RCP 8.5 and the MIROC5 climate model.
