# Supplementary material for: Historical and projected future range sizes of the world’s mammals, birds, and amphibians
Source: Nat Commun. 2020 Nov 6;11:5633. doi: 10.1038/s41467-020-19455-9 (PMC7648644; doi:10.1038/s41467-020-19455-9)
Supplement: Supplementary file 17 — Reporting Summary [file 41467_2020_19455_MOESM17_ESM.pdf]

nature research

Corresponding author(s): Dr Robert Beyer

Last updated by author(s): Oct 9, 2020

Reporting Summary

Nature Research wishes to improve the reproducibility of the work that we publish. This form provides structure for consistency and transparency in reporting. For further information on Nature Research policies, see our Editorial Policies and the Editorial Policy Checklist.

Statistics

For all statistical analyses, confirm that the following items are present in the figure legend, table legend, main text, or Methods section.

n/a	Confirmed	
☐	☒	The exact sample size (n) for each experimental group/condition, given as a discrete number and unit of measurement
☐	☒	A statement on whether measurements were taken from distinct samples or whether the same sample was measured repeatedly
☐	☒	The statistical test(s) used AND whether they are one- or two-sided<br>*Only common tests should be described solely by name; describe more complex techniques in the Methods section.*
☒	☐	A description of all covariates tested
☒	☐	A description of any assumptions or corrections, such as tests of normality and adjustment for multiple comparisons
☒	☐	A full description of the statistical parameters including central tendency (e.g. means) or other basic estimates (e.g. regression coefficient) AND variation (e.g. standard deviation) or associated estimates of uncertainty (e.g. confidence intervals)
☐	☒	For null hypothesis testing, the test statistic (e.g. F, t, r) with confidence intervals, effect sizes, degrees of freedom and P value noted<br>*Give P values as exact values whenever suitable.*
☒	☐	For Bayesian analysis, information on the choice of priors and Markov chain Monte Carlo settings
☒	☐	For hierarchical and complex designs, identification of the appropriate level for tests and full reporting of outcomes
☒	☐	Estimates of effect sizes (e.g. Cohen's d, Pearson's r), indicating how they were calculated

Our web collection on statistics for biologists contains articles on many of the points above.

Software and code

Policy information about availability of computer code

Data collection	No software was used to collect data
Data analysis	Matlab R2019a and R v3.6.3. Code associated with this study is available at: https://doi.org/10.17605/OSF.IO/TJ6C5

For manuscripts utilizing custom algorithms or software that are central to the research but not yet described in published literature, software must be made available to editors and reviewers. We strongly encourage code deposition in a community repository (e.g. GitHub). See the Nature Research guidelines for submitting code & software for further information.

Data

Policy information about availability of data

All manuscripts must include a data availability statement. This statement should provide the following information, where applicable:

- Accession codes, unique identifiers, or web links for publicly available datasets
- A list of figures that have associated raw data
- A description of any restrictions on data availability

Data associated with this study are available on the Open Science Framework: https://doi.org/10.17605/OSF.IO/TJ6C5. Bird distribution and habitat data are available from: http://datazone.birdlife.org/species/requestdis; mammal and amphibian distribution and habitat data are available from: https://www.iucnredlist.org/; HYDE 3.2 past land use reconstructions are available from: https://doi.org/10.17026/dans-25g-gez3; AIM future land use projections: https://doi.org/10.7910/DVN/4NVGWA; Historical and future climate simulations are available from: https://esgf-node.llnl.gov/search/cmip5/;1901–2016 observed climate data are available from: https://doi.org/10.5285/10d3e3640f004c578403419aac167d82.

Field-specific reporting

Please select the one below that is the best fit for your research. If you are not sure, read the appropriate sections before making your selection.

☐ Life sciences ☐ Behavioural & social sciences ☒ Ecological, evolutionary & environmental sciences

For a reference copy of the document with all sections, see nature.com/documents/nr-reporting-summary-flat.pdf

Ecological, evolutionary & environmental sciences study design

All studies must disclose on these points even when the disclosure is negative.

Study description	Estimation of historical and projected future range sizes of mammals, birds, and amphibians
Research sample	Global mammal, bird, and amphibian species (species distribution and habitat data are available at http://datazone.birdlife.org/species/requestdis and https://www.iucnredlist.org/)
Sampling strategy	This study did not involve sampling
Data collection	This study did not involve collection of new data
Timing and spatial scale	Year 1700 - 2100, Global
Data exclusions	No data were excluded
Reproducibility	This study did not involve experiments
Randomization	This study did not involve randomisation (datasets and code are deterministic)
Blinding	This study did not involve blinding

Did the study involve field work? ☐ Yes ☒ No

Reporting for specific materials, systems and methods

We require information from authors about some types of materials, experimental systems and methods used in many studies. Here, indicate whether each material, system or method listed is relevant to your study. If you are not sure if a list item applies to your research, read the appropriate section before selecting a response.

Materials & experimental systems

n/a	Involved in the study
☒	☐ Antibodies
☒	☐ Eukaryotic cell lines
☒	☐ Palaeontology and archaeology
☒	☐ Animals and other organisms
☒	☐ Human research participants
☒	☐ Clinical data
☒	☐ Dual use research of concern

Methods

n/a	Involved in the study
☒	☐ ChIP-seq
☒	☐ Flow cytometry
☒	☐ MRI-based neuroimaging
